# Building Finite Element Models to Investigate Zebrafish Jaw Biomechanics

**DOI:** 10.3791/54811

**Published:** 2016-12-03

**Authors:** Lucy H. Brunt, Karen A. Roddy, Emily J. Rayfield, Chrissy L. Hammond

**Affiliations:** ^1^Physiology, Pharmacology and Neuroscience, University of Bristol; ^2^Earth Sciences, University of Bristol

**Keywords:** Developmental Biology, Issue 118, Zebrafish, Biomechanics, Strain, Musculoskeletal, Finite Element, Confocal, Morphology, Joint morphogenesis

## Abstract

Skeletal morphogenesis occurs through tightly regulated cell behaviors during development; many cell types alter their behavior in response to mechanical strain. Skeletal joints are subjected to dynamic mechanical loading. Finite element analysis (FEA) is a computational method, frequently used in engineering that can predict how a material or structure will respond to mechanical input. By dividing a whole system (in this case the zebrafish jaw skeleton) into a mesh of smaller 'finite elements', FEA can be used to calculate the mechanical response of the structure to external loads. The results can be visualized in many ways including as a 'heat map' showing the position of maximum and minimum principal strains (a positive principal strain indicates tension while a negative indicates compression. The maximum and minimum refer the largest and smallest strain). These can be used to identify which regions of the jaw and therefore which cells are likely to be under particularly high tensional or compressional loads during jaw movement and can therefore be used to identify relationships between mechanical strain and cell behavior. This protocol describes the steps to generate Finite Element models from confocal image data on the musculoskeletal system, using the zebrafish lower jaw as a practical example. The protocol leads the reader through a series of steps: 1) staining of the musculoskeletal components, 2) imaging the musculoskeletal components, 3) building a 3 dimensional (3D) surface, 4) generating a mesh of Finite Elements, 5) solving the FEA and finally 6) validating the results by comparison to real displacements seen in movements of the fish jaw.

**Figure:**
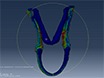


## Introduction

Finite Element (FE) modelling is an engineering technique that can computationally calculate and map the magnitude and location of strains acting on a structure ^1^. The model consists of the 3D structure, represented by a mesh of "Finite Elements", and the end result of the analysis is governed by a number of factors including the structure and number of elements in the mesh, the magnitude and location of the mechanical loads and the material properties. Material properties describe certain aspects of a material's behavior under a given type of load; Young's modulus (E) describes the elasticity of the material while Poisson's ratio describes the proportional decrease in the width of a material to its length when a sample is stretched. FE modelling can be used to calculate a variety of variables including displacement, stress, pressure and strain acting on the model by taking into account the unique input data about the structure's shape, location and magnitude of loads and the specific material properties.

FE modeling is widely used in engineering ^2^ and increasingly for orthopedic ^3^ and paleontological applications^4^. In development biomechanical forces are known to act as a stimulus in many cells to activate cell responses ^5-8^ and it is useful to predict both the relative positions and magnitudes of mechanical stimuli within developing organ systems, however, currently FE modeling has been little used for zebrafish development.

Both cartilage and bone have been shown to be mechanosensitive materials. For example, *in vitro *compression has been found to activate chondrogenic pathways, whilst tension has been shown to be necessary for bone formation ^9^. FE analysis (FEA) has been exploited to model strains acting on biological specimens, including those acting on skeletal elements during bone formation ^10^. Other development applications include its use to predict the shape of a joint after it has been exposed to theoretical biomechanical forces ^11,12^ and to show the pattern of strains present during chick knee joint morphogenesis ^8^.

This protocol is aimed at sharing the experience of generating 3-dimensional surfaces, meshes and Finite Element models from confocal images with a view to understanding the mechanics of developing tissues. We also show ways of validating the FE models though capturing real joint displacement information *in vivo*. While we use the zebrafish jaw as an exemplar the same techniques could be used on any small biological system for which 3D information on the structure of the musculoskeletal system can be obtained by confocal or multiphoton imaging.

## Protocol

All steps within the protocol follow the University of Bristol's animal care and welfare guidelines and those of the U.K. Home Office.

### 1. Visualization of Musculoskeletal Anatomy

NOTE: To visualize the shape of the skeletal elements, to quantify muscle and to identify the exact placement of the muscle attachments, immunostain (section 1.1) fish at the appropriate age for skeletal myosin (which reveals muscle) and type II collagen (to visualize cartilage). Alternatively, visualize the musculoskeletal anatomy using transgenic fluorescent reporter lines such as the collagen a1 reporter col2a1:mCherry ^13,14^ to visualize cartilage and the slow myosin heavy chain reporter smyhc:GFP ^15^ to visualize the position of muscle attachments (Section 1.2).

Alternative lines that mark cartilage and muscle could work equally well.

1. **Fluorescent Immunostaining**
  1. Fix larva in excess 4% paraformaldehyde (PFA) in phosphate-buffered saline (PBS) for 1 hr. Wash in PBS with 0.1% Tween 20 (PBT) and dehydrate in 50% methanol (MeOH) in PBT and 100% MeOH for 5 min respectively. Caution: PFA is toxic and should be handled in accordance with the material safety sheet. NOTE: Larva can be stored in 100% MeOH until required.
  2. Rehydrate larva in 50% MeOH in PBT for 5 min. Wash in PBT for 5 min.
  3. Permeabilize larva in 0.25% trypsin in PBT on ice for 5-6 min. Wash in 4x PBT for 5 min each.
  4. Block for 2-3 hr in 5% serum in PBT.
  5. Incubate larva in the recommended dilution of rabbit anti-type 2 collagen and mouse anti-myosin antibodies in 5% serum in PBT for 1 hr at room temp or overnight at 4 °C. NOTE: The recommended dilution range is normally on the antibody data sheet. Choose antibodies that are raised against different species to one another and that are also different to the tissue.
  6. Wash larva 6x for 15 mins in PBT.
  7. Block for 1-2 hr in 5% serum in PBT.
  8. Incubate in secondary antibodies in the dark. Use fluorescently labelled anti-mouse (550) and anti-rabbit (488) secondary antibodies at an appropriate dilution in 5% serum and PBT for the specific antibody.
  9. Wash 6X for 10 minutes each in PBT and image on a 10X confocal microscope as soon as possible.
2. **Imaging Musculoskeletal Geometry**
  1. Mount the larvae ventrally on a coverslip in tepid 0.3-0.5% low melting point (LMP) agarose in Danieau's solution ^16^. NOTE: Transgenic fish will need to be sedated in 0.02% MS222 (Tricaine methanesulfonate, pH 7) before mounting and during imaging.
  2. Take a confocal image stack of the region of interest using the 10X objective lens and the approximately 2.5X digital zoom. Prepare images of the green and red channel using the 488 nm and 561 nm lasers respectively. Image at a 512 x 512 pixel resolution with an interval between z planes of 1.3 µm and 3 line averages. The resulting stack will comprise of approximately 100 z slices.
  3. Export the data as a tiff series. Maximum projections of the muscle and cartilage elements from a 5dpf zebrafish larva are shown in **Figure 1**.

### 2. Generating a 3D Surface

1. Choose a representative dataset for each time point at 3, 4 and 5 dpf (choose after visualization of multiple samples).
2. Open 3-dimensional tiff stack and select all channels in analysis software. Right click on the cartilage channel and select image filter and smoothing:Gaussian (**Figure 2B**).
3. In project view right click on filtered image and select 'image segmentation' and then 'edit new label'. Create a new label for each material, *i.e.,* cartilage and joint. Select the cartilage region of the image (**Figure 2C**, white signal, purple outline) using the magic wand tool. Use the brush tool to remove noise from the outlines. NOTE: If using magic wand tool, click 'All slices'.
4. Select the joint region with the brush tool and assign to a joint component (**Figure 2C**, blue outline)
5. Smooth multiple slices at once by selecting segmentation in the top menu and smooth labels. Right click on the image and select generate surface to produce a 3D surface render of the component (**Figure 2D**).
6. Click on the surface and save data as a hmascii file for import into meshing software.

### 3. Calculating the Muscle Forces to Be Used in the FE Model

1. Count the number of muscle fibers from confocal images of smyhc:GFP transgenic zebrafish (**Figure 1A**, arrowhead, 1C) and measure the diameter of the fibers to calculate their cross sectional area (πr^2^).
2. Identify suitable force per muscle unit area from the literature. The maximal muscle force generated per unit area for larval zebrafish skeletal muscles (40 nN/µm^2^) were used ^17^.
3. Calculate the forces for each anatomical muscle group by multiplying the number of fibers and their area by the force per unit area. See **Table 1**.

### 4. Generating a Mesh

1. Import the 3D model generated in section 2 (above) into a software package capable of generating a finite element mesh.
2. Generate a2D mesh of the cartilage and joint surfaces by using the shrink wrap tool under the 2D menu. Choose an appropriate element size. Note: Use an element size between 1.5-2.5. If necessary, generate a range of differently sized 2D surface meshes to carry out 3D mesh optimization (Section 4.4).
3. Carry out mesh quality checks found under the '2D>Tools>Check elements' panel to check for duplicated elements, insertions and penetrations in the mesh. Fix dihedral angles by using the utility tab in model tree.
4. Generate a 3D mesh from the 2D surface meshes of differing element size using the 3D>Tetramesh subpanel. NOTE: Compare the results of different mesh sizes and select the FE model with the lowest mesh size that converges after further simulations and does not compromise feature definition. The example in **Figure 3** contains 1.5 million tetrahedral elements for the lower jaw cartilages and had a 2D element size of 2.0.
5. Transform the mesh so jaw model is to scale as per confocal stack using the Geometry>Distance subpanel. NOTE: Ensure the cartilage and joint components are connected in the model by exporting a merged model or by using ties.

### 5. Finite Element Model Construction

1. Using commercial finite element (FE) software, create a FE model. Using the 3D muscle and cartilage labeled confocal stacks generated in section 1 as a guide, assign nodes that correspond to muscle attachment points. Create a vector between two nodes representing the origin and insertion of each muscle (**Figure 3**).
2. Create a load collector of type 'history' to apply a 'C load' for each muscle. Specify the magnitude in Newtons (calculated in step 3.3) and assign the associated vector. **Figure 3** shows the attachment points for the adductor mandibulae (AM), protractor hyoideus (PH) and intermandibularis (IM). NOTE: For these jaw muscles, maximum contractile force is distributed between the origin and insertion so that only 50% of each load is applied at each site.
3. Assign appropriate elastic isotropic material properties as determined by the literature. Young's modulus for cartilage and the interzone in this model were 1.1 MPa and 0.25 MPa respectively and Poisson's ratio was 0.25 for both^ 18,19^.
4. Create a load collector of type 'boundary' to apply initial constraints on the model. Go to the tab Analysis>Constraints and in the create subpanel, pick the nodes on the model you wish to constrain. Select the degrees of freedom (DOF) that limit movement of the model to the best approximation of its natural range of motion. NOTE: The model in **Figure 3** was constrained in all axes of motion (DOF: 1, 2, 3 represent x, y and z, respectively) at the ceratohyal to anchor it in space at a mid-point in the model and in the y and z axis at the point where the palatoquadrate connects to the rest of the zebrafish skull (**Figure 3**, **Table 1**). The model must be constrained in all three DOF in a least one node.
5. Create a 'Load step', for each type of movement you wish to simulate (*i.e.,* opening, closing), under the analysis menu and select all the relevant loads (made in Section 5.2) and constraints (made in Section 5.4) to simulate this movement. Select 'Static' from the drop down menu when it appears.
6. Export model including the mesh, loads, constraints and material properties in an appropriate file format, in this case ".inp" format.
7. Load model into FE analysis software. Create and execute a job for the model using the Job module.
8. Analyze output for stress, strain, displacement, *etc.* found in the results tab and visualization menu (**Figure 4** and **5**).

### 6. Validation of Jaw Deformation/Displacement Distances

1. Select 3-6 *Tg(Col2a1aBAC:mcherry)* transgenic zebrafish.
2. Lightly anaesthetize the larvae with 0.02% MS222 until they cease to respond to touch but their hearts are still beating.
3. Mount the larvae laterally (while anaesthetized) on coverslips in tepid 1% LMP agarose (made up in Danieau's solution).
4. Remove the agarose from around the head and jaw with forceps.
5. Flush fresh Danieau's solution (with no MS222) over the head of the larva to remove anesthesia using a Pasteur pipette until normal mouth movements resume.
6. Use movie capture software to take bright-field high-speed videos of mouth movements. Take movies of around a minute duration at the highest frame rate, or sufficient to record multiple cycles of jaw opening.
7. Choose frames that show the jaw open to its maximum displacement. Measure the distance between the anterior tip of the Meckel's cartilage and the upper jaw (tip of ethmoid plate) in µm.
8. Calculate the average displacement from multiple fish larvae.
9. Extract displacement data from the model. Use the average displacement calculated in 6.8 to verify model displacement behavior (**Figure 4**). If the model deviates from expected, run sensitivity analysis by sequentially altering material properties and muscle loads.

## Representative Results

Immunostaining for muscle (**Figure 1A**) and cartilage (**Figure 1B**) or imaging of transgenic reporters (**Figure 1C**) allows the 3D structure of the jaw to be visualized, along with the associated musculature. By imaging at a high resolution it was possible to build a model that captures both the three-dimensional shape of the jaw (**Figure 2**) and the location and placement of loads (**Figure 3**). Utilizing *in vivo* displacements seen through high speed video capture (**Figure 4**) we verified that the range of motion in the model was within a realistic range.

The FE models once run can be used to display a range of data, such as stress (**Figure 5A**), minimum and maximum principal strain (**Figure 5B**-**K**). These results are three-dimensional so the model can be magnified to see fine patterns of detail (**Figure** **5E**, **5I**) rotated to obtain relevant views (**Figure 5F**, **5G**, **5J**, **5K**) and digitally sectioned (**Figure 5E'**, **5E''**, **5I'**, **5I''**) to show how the patterns of stress, strain or pressure change throughout the model. It is also possible to extract quantitative data from the model (not shown). By verifying the model and using the most accurate material properties, loads and mesh shape the FE model can be used to explore the best estimate of the mechanical environment being experienced by cells during that window of development. The results of the model can be directly compared to changes in cellular behavior and gene expression^ 20^.


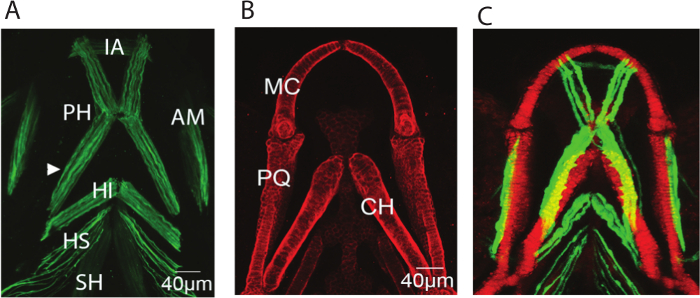
**Figure 1:Representative images of the musculoskeletal elements of the zebrafish lower jaw at 5 dpf. **Representative confocal stacks of the lower jaw of 5dpf larvae all shown with anterior to top (**A**) Immunostaining for A4.1025 which stains all skeletal myosin (**B**) Immunostaining for Type II collagen which marks all cartilage (**C**) Stack from a live larva expressing the transgenic reporters col2a1:mCherry marking cartilage (red) and smyhc:GFP slow muscle (green). IA: intermandibularis anterior, PH: protractor hyoideus, AM:adductor mandibulae, HI: hyoideus inferior, HI: hyoideus superior, CH: sternohyoideus, MC: Meckel's cartilage, PQ: Palatoquadrate, CH: ceratohyal. Please click here to view a larger version of this figure.


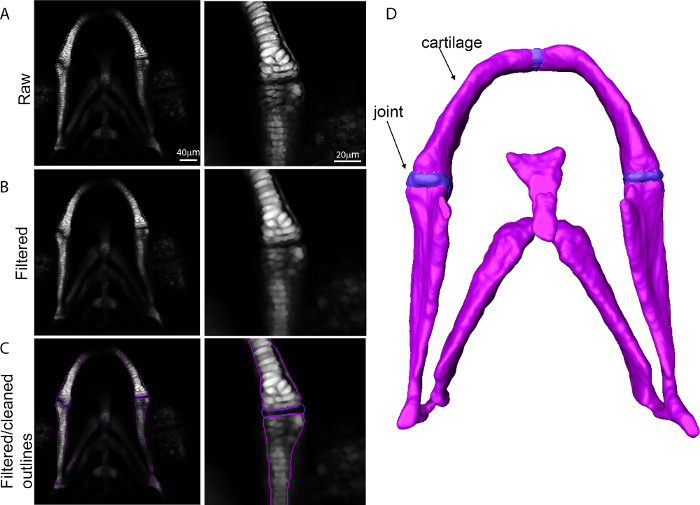
**Figure 2:Generation of a 3D surface from confocal data. **Images showing the transition from confocal data into a 3D surface for the zebrafish lower jaw with higher magnification of the joint region. (**A**) Raw confocal data; (**B**) Dataset after application of a Gaussian filter; (**C**) Filtered outline; (**D**) 3D surface. Please click here to view a larger version of this figure.


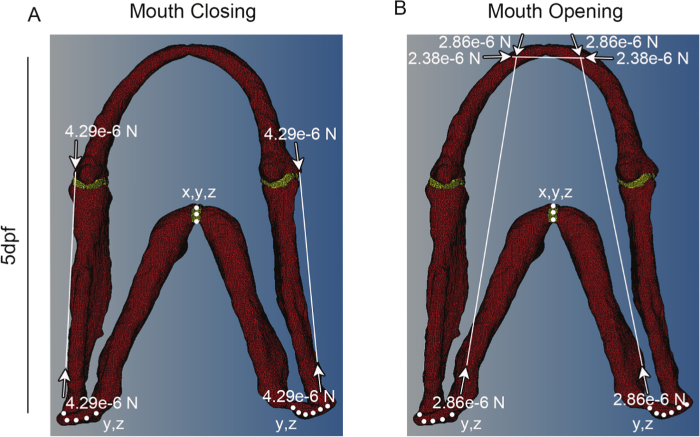
**Figure 3:Representative meshes showing constraints and force vectors. **Representative meshes for a 5 dpf larva for (**A**) mouth closing and (**B**) mouth opening. White dots denote places where the model is constrained and in which dimensions (*e.g., *x and y or x, y and z). White lines denote muscle positions, with the vector of muscle force denoted by white arrows. Red shows cartilage and Yellow the interzone. This figure has been modified from supplementary material previously published in Brunt *et al.*^ 15^. Please click here to view a larger version of this figure.


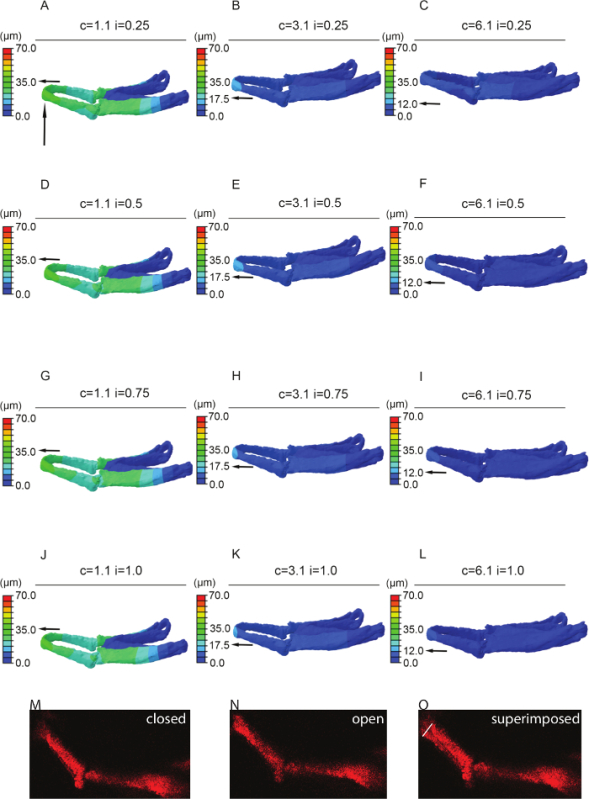
**Figure 4:Sensitivity testing. **FE-model simulating jaw displacement in 5dpf zebrafish for different cartilage and interzone Young's moduli. Jaw displacement (open to closed in µm) is marked on the jaw; recorded using the color key. Each model (**A**-**L**) has a different combination of cartilage (c = 1.1, 3.1, or 6.1 MPa) or interzone (i = of 0.25, 0.5, 0.75, or 1 MPa) properties. Horizontal black arrow highlights the value of jaw displacement at the tip of the Meckel's cartilage (represented by the vertical black arrow). **M** and **N** stills from videos of 5 dpf larvae showing minimum*, i.e., *jaw closed (**M**) and maximum, *i.e.,* jaw fully open (**N**) with the two superimposed (**O**) - white line on O represents the displacement (of 43 µm). In this case relative cartilage properties of 1.1 with an interzone of 0.25 (A) best match the displacements seen in live fish (**O**). Panels A-L of this figure have been previously published in Brunt *et al.*^ 15^. Please click here to view a larger version of this figure.


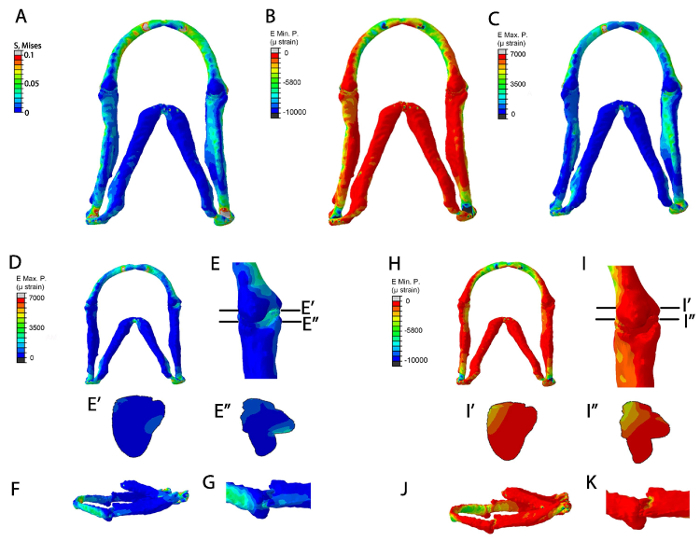
**Figure 5: Representative data from the FE models. **FE-model simulation of all muscles applied in a 5 dpf larva (**A**-**C**). (**A**) Von Mises (EMaxmin) (**B**) Minimum Principal strain (E Min. P, µɛ) (**C**) Maximum principal strain (E Max. P., µɛ). FE-model simulation of maximum and minimum principal strains during jaw opening. (**D**-**K**): Maximum principal strain (E Max. P., µɛ) in (**D**) ventral jaw view and (**E**) ventral joint view (**E**) shows location of proximal-distal sections through the Meckel's cartilage joint and the interzone in (**E'**) and (**E''**), respectively. (**F**): lateral jaw view. (**G**): lateral joint view. (**H**-**K**): Minimum Principal strain (E Min. P, µɛ) in (**H**) ventral jaw view and (**I**) ventral joint view. (**I**) shows location of the proximal-distal sections through the Meckel's cartilage joint and interzone in (**I'**) and (**I''**) respectively (**J**): lateral jaw view. (**K**): lateral joint view. This figure has been previously published in Brunt *et al.*^ 15^. Please click here to view a larger version of this figure.

**Table:** 

	Number of muscle fibers	Muscle fiber area (µm^2^)	Muscle group area (µm^2^)	Force (N)
5 dpf intermandibularis anterior	5	23.8	119	4.76e-6
5 dpf protractor hyoideus	6	23.8	142.8	5.71e-6
5 dpf adductor mandibulae	9	23.8	214.2	8.57e-6

**Table 1:Muscle quantification. **Calculated average muscle forces for the Intermandibularis Anterior, Adductor Mandibulae and Protractor Hyoideus at 5 dpf using 40 nN/µm^2^ (value per unit area taken from reference^ 17^). (Lorga *et al.*, 2011) (n = 3).

## Discussion

Finite Element models have been used to relate the areas of skeletal elements that are under strain with those undergoing bone formation ^10^, as well as to map the areas under strain during endochondral ossification and joint morphogenesis ^8,12,21^. Other studies have also been able to apply theoretical growth models to replicate changes during joint development ^11,12^. Here we show the protocol for building FE models for a relatively simple system, the zebrafish jaw ^20^. Unlike alternative methods of collecting raw images for the FE models, such as CT scanning^ 22^, confocal imaging of transgenic lines or immunostained zebrafish allows for multiple tissues to be studied. It can, therefore, provide direct information on muscle attachment points in relation to cartilage. Among vertebrate models zebrafish are particularly amenable to genetic and pharmacological manipulation. The generation of FE models for zebrafish craniofacial cartilage now opens up the possibility of further study of the interplay between biomechanics and genetics in joint morphogenesis.

There are a number of critical steps to the process of creating an FE model; the first is generating an accurate three-dimensional representation of the system. This requires imaging at high enough resolution to clearly define boundaries. Note that even with high-resolution imaging to make a good surface one may have to smooth out some regions. Another critical step is defining the correct placement of the load and correct constraints. An insufficiently constrained model will fail to solve and incorrect placement of the loads will cause abnormal movement.

Some processing of the raw data (**Figure 2**) is necessary as a surface generated from the raw data would be difficult to mesh (**Figure 2B**). We filtered the data using a Gaussian filter (**Figure 2C**) and we carried out some manual smoothing of the curves to produce a set of clean outlines that can be converted into a 3D surface. Too much smoothing can produce a "melted" surface that has lost many of its features. Choosing the correct element size is an iterative process as choosing too small an element size creates too large a mesh which is computationally intensive. However, choosing too large an element size will produce a mesh which fails to recapitulate the correct shape of the structure. The correct mesh had the smallest element size that captured the correct shape of the jaw and converged on a correct solution, verified using the jaw displacement. It may also be necessary to modify the material properties or load calculations to better emulate the correct displacement as different ages and species will have substantially different properties.

It is important to remember that there are always limitations to a hypothetical model and assumptions made to run FE models. When only modeling one or a small number of samples it is critical to ensure that a representative sample is chosen as there are likely to be small variations between individuals. As only some of the jaw elements and muscles were included, the model is a simplified version of the zebrafish craniofacial musculoskeletal system. Therefore, constraints had to be positioned to account for where the modelled jaw elements would connect with the rest of the skull and the model was artificially constrained in the center to fix it in 'space'. This artificial constraint did not impact on the interpretation drawn from the models as the ceratohyal itself was not analyzed. The inclusion of more of the craniofacial structure, especially other jaw opening muscles such as the sternohyals and its attached cartilage ^23^, could have added to the model, but limitations include the ability of larger models to run in the Finite Element software.

Another limitation is that we have not modeled ligament insertion, though this could be achieved by the insertion of springs ^8^. One other assumption made in this case was that the model would behave linearly. The magnitudes of strains on the models were comparable to those in published models and applied to *in vitro *cells ^10,24^, with strains being below +3,500 and above -5,000 µɛ apart from constraint and muscle attachment points. Therefore, the strains at the relevant regions of the model were deemed within a range acceptable for a linear model. Cartilage does not behave entirely as a linear material and has previously been modelled as a poroelastic material, which enabled analysis of the fluid behavior in the model ^25^. Spreading the muscle attachment points amongst a cluster of local nodes would distribute the peak forces and more accurately represent the muscle insertion for certain muscles.

Use of FE allows an assessment of the strains and stresses acting on a structure. As a technique it is frequently used in many bioscience disciplines including orthopedics, paleontology and more recently developmental biology. Here we describe how to build FEs for the zebrafish lower jaw. In the future these models could be extended to look at the whole jaw, including the palate. Similar techniques could be used to model spinal biomechanics in fish, which to date have mostly been studied by kinematic means.

## Disclosures

The authors have nothing to disclose.

Some data in Figs 3-5 has been reprinted from J.Biomech, 48 (12), Brunt *et al.*, Finite element modeling predicts changes in joint shape and cell behavior due to loss of muscle strain in jaw development, 3112-22., 2015, with permission from Elsevier^15^.
